# Genetic Background Modulates Zoliflodacin and Gepotidacin Cross-Resistance and Fitness in *Neisseria gonorrhoeae*

**DOI:** 10.1093/infdis/jiag174

**Published:** 2026-03-19

**Authors:** Aditi Mukherjee, Sofia O P Blomqvist, David Helekal, Apabrita A Das, Samantha G Palace, Yonatan H Grad

**Affiliations:** Department of Immunology and Infectious Diseases, Harvard T. H. Chan School of Public Health, Boston, Massachusetts, USA; Department of Immunology and Infectious Diseases, Harvard T. H. Chan School of Public Health, Boston, Massachusetts, USA; Department of Immunology and Infectious Diseases, Harvard T. H. Chan School of Public Health, Boston, Massachusetts, USA; Cardiovascular Medicine Division, Brigham and Women's Hospital, Boston, Massachusetts, USA; Harvard Medical School, Boston, Massachusetts, USA; Department of Immunology and Infectious Diseases, Harvard T. H. Chan School of Public Health, Boston, Massachusetts, USA; Department of Immunology and Infectious Diseases, Harvard T. H. Chan School of Public Health, Boston, Massachusetts, USA

**Keywords:** *Neisseria gonorrhoeae*, zoliflodacin, gepotidacin, cross-resistance

## Abstract

**Background:**

The emergence of multidrug-resistant *Neisseria gonorrhoeae* has created an urgent need for new therapeutic options. Zoliflodacin and gepotidacin, 2 first-in-class topoisomerase inhibitors, are oral antibiotics recently approved by the FDA for gonorrhea treatment. Resistance to zoliflodacin can occur through *gyrB*^D429N^, but this mutation's impact on fitness and on resistance to other topoisomerase-targeting drugs have been unclear. The aim of this study was to investigate the in vitro fitness and antibiotic resistance implications of *gyrB*^D429N^ in ciprofloxacin*-*resistant *N. gonorrhoeae* strains.

**Methods:**

We introduced the *gyrB*^D429N^ substitution into 9 clinical strains via transformation. Antimicrobial susceptibility testing was performed for zoliflodacin, gepotidacin, and ciprofloxacin using standard MIC assays. In vitro relative fitness of parent and resistant mutant strains was assessed by pairwise competition experiments.

**Results:**

*gyrB* ^D429N^ conferred cross-resistance to gepotidacin in 3 clinical strains, and its fitness effect varied by strain background. In particular, *parC*^D86N^ appeared to potentiate *gyrB*^D429N^ cross-resistance to gepotidacin, a drug for which resistance has previously only been seen in the presence of both *parC*^D86N^ and *gyrA*^A92T^ mutations.

**Conclusions:**

Genetic background modulated the phenotypic effects of a zoliflodacin-resistance determinant on fitness and cross-resistance to gepotidacin. These findings inform strategies for introducing the new topoisomerase inhibitors into clinical use and for surveillance of resistance.

## Teaser

Genetic background shapes the impact of the zoliflodacin-resistance mutation *gyrB*^D429N^ on gepotidacin-susceptibility in *Neisseria gonorrhoeae*, revealing a potential cross-resistance pathway among new topoisomerase inhibitors.

*Neisseria gonorrhoeae*, the causative agent of the sexually transmitted infection gonorrhea, has developed resistance to all antibiotics used for its treatment [1]. The World Health Organization estimated over 82 million new *N. gonorrhoeae* infections in 2020, with the highest burden of antimicrobial resistance reported in Asia [2]. The prevalence of ciprofloxacin resistance in Asia is at or near 100% [3], and ceftriaxone resistance has reached 30% in some regions [4]. These trends accelerated efforts to develop novel antimicrobial agents with distinct mechanisms of action and minimal cross-resistance with existing drugs. Two new, first-in-class antibiotics, zoliflodacin and gepotidacin, successfully completed phase 3 trials for uncomplicated urogenital gonorrhea [5, 6], have recently been approved by the FDA [7], and are awaiting regulatory review in other jurisdictions. Importantly, zoliflodacin and gepotidacin represent structurally distinct antimicrobial classes from fluoroquinolones despite also targeting type II topoisomerases.

Like ciprofloxacin, zoliflodacin and gepotidacin target type II topoisomerases. Ciprofloxacin, a fluoroquinolone, inhibits the A subunit of DNA gyrase (*gyrA*) and A subunit of topoisomerase IV (*parC*) [8]. Resistance to ciprofloxacin arises from mutations in codons 91 and 95 in *gyrA* and 86–91 in *parC* [9]. In contrast, zoliflodacin, a spiropyrimidinetrione, targets the *gyrB* subunit of DNA gyrase [10]. Zoliflodacin resistance has not been observed in common circulating gonococcal lineages, likely because this drug has not yet been deployed for gonorrhea treatment. In laboratory settings, zoliflodacin resistance has been linked to substitutions in *gyrB*, including D429N, K450N, and K450T [11, 12]. Additionally, the S467N mutation in *gyrB* appears to act as a stepping-stone mutation to zoliflodacin resistance but does not itself confer resistance to zoliflodacin [13].

Gepotidacin, a triazaacenaphthylene, targets both the A subunit of DNA gyrase (*gyrA*) and A subunit of topoisomerase IV (*parC*) [8]. In gepotidacin's phase 2 clinical trial for treatment of gonorrhea, resistance arose via spontaneous *gyrA*^A92T^ mutation in 2 strains that harbored *parC*^D86N^, which also contributes to ciprofloxacin resistance [14–17]. Spontaneous resistance was not observed in the phase 3 trial for gepotidacin treatment of gonorrhea, likely as a result of the switch from the single dose used in the phase 2 trial [6] to 2 doses given 10–12 hours apart.

Zoliflodacin and gepotidacin are active against ciprofloxacin-resistant *N. gonorrhoeae* and cross-resistance among these 3 topoisomerase-targeting drugs has not been reported in a clinical setting [17, 18]. However, the overlapping targets of these drugs raises the concern that cross-resistance is possible, as has been seen to a limited extent for the *gyrB*^D429N^ mutation in the context of increasing ciprofloxacin resistance [11]. It is particularly important to understand if existing genetic diversity in topoisomerase components among prevalent ciprofloxacin-resistant lineages may increase the likelihood of acquiring resistance to zoliflodacin and gepotidacin, either by enabling cross-resistance or by ameliorating resistance-associated fitness costs. Here, we investigated this potential issue, focusing on the zoliflodacin-resistance mutation *gyrB*^D429N^ that is known to influence susceptibility to nalidixic acid [19] and ciprofloxacin [11]. To determine how phenotypes conferred by this mutation are influenced by genetic background, we examined the effect of *gyrB*^D429N^ on cross-resistance to other replisome-targeting drugs and on competitive fitness in a panel of clinical strains representing circulating *N. gonorrhoeae* strains in Asia.

## METHODS

### Phylogenetic Analysis

We assembled a dataset of 2800 published genomes [20–23] with a subset of the genomes collected by the Centers for Disease Control and Prevention's (CDC) 2024 surveillance efforts. To obtain the genomes of the CDC strains, we searched the NCBI Pathogen Detection database (https://www.ncbi.nlm.nih.gov/pathogens/) for *N. gonorrhoeae* strain genome sequences collected by the US CDC with a collection date between 1 January 2024 and 31 December 2024 (“Home—pathogen detection—NCBI”). We filtered out strains that were not identified as a GCWGS strain, the designation given to strains collected in the context of CDC *N. gonorrhoeae* surveillance. *N. gonorrhoeae* sequence typing for antimicrobial resistance (NG-STAR) and multi-locus sequencing typing (MLST) for strains used for additional experiments are included in Supplementary Table 1.

Reference-based mapping to NCCP11945 (NC_011035.1) was done using BWA-MEM v0.7.17 [24]. We used Pilon v1.23 to call variants (minimum mapping quality: 20, minimum coverage: 10X) [25] after marking duplicate reads with Picard v2.20.1 (https://broadinstitute.github.io/picard/) and sorting reads with SAMtools v1.17 [26]. We generated pseudogenomes by incorporating variants supported by at least 90% of reads and sites with ambiguous alleles into the reference genome sequence. The pseudogenomes were then used as the input alignment for subsequent phylogenetic reconstruction.

We used GUBBINS v3.4.3 [27] to estimate recombining regions and IQTREE v2.4.0 [28] for phylogenetic reconstruction. We used MODELFINDER [29] to select an optimal molecular clock model. The phylogenetic tree was visualized using iTOL v7 [30].

We generated pseudogenomes and used these to call *gyrA* and *parC* variants as described above for strains published previously [31, 32].

### Molecular Docking

Protein sequences of *N. gonorrhoeae* GyrA and GyrB were retrieved from UniProt (accessions P48371 and P22118, respectively). Tertiary structures of GyrA and GyrB monomers were predicted independently using ColabFold v1.5 [33] with AlphaFold2-multimer presets and default parameters. For each monomer, the top-ranked model was selected from ColabFold results. GyrA and GyrB monomers were docked with ClusPro 2.0 protein-protein docking server [34] using balanced scoring model and the highest ranked structure was used for structural analysis. ChimeraX v1.10.1 [35] was used for visualization of protein interfaces.

### *N. gonorrhoeae* Culture Conditions

*Neisseria gonorrhoeae* was cultured on GCB agar (Difco) supplemented with Kellogg's supplement (GCB-K) at 37°C with 5% CO_2_ as previously [36]. Growth curve and pairwise competition experiments were conducted in liquid GCP medium containing 15 g/L proteose peptone 3 (Thermo Fisher), 1 g/L soluble starch, 1 g/L KH_2_PO_4_, 4 g/L K_2_HPO_4_, and 5 g/L NaCl (Sigma-Aldrich) with Kellogg's supplement with agitation at 37°C with 5% CO_2_.

### Generation of Isogenic *N. gonorrhoeae gyrB* Mutant Strains

Strains, plasmids and primers used in this study are listed in Supplementary Table 1.

The mutant *gyrB*^D429N^ allele was amplified using primers AM_1 (F) and AM_2 (R) from the genomic DNA of a previously reported experimentally evolved *N. gonorrhoeae* GCGS0481 strain that acquired the *gyrB*^D429N^ mutation [11] and introduced into the selected strains by electroporation [36]. Individual colonies were selected on GCB-K plates from within the zone of inhibition created by a dried droplet of 4 μg/mL zoliflodacin. Transformants were verified by Sanger sequencing of the *gyrA* and *gyrB* loci and by whole genome sequencing. Genomic DNA from parent and mutant strains were purified using an Invitrogen PureLink Genomic DNA Mini Kit (K182001), prepared for sequencing using Oxford Nanopore Technologies Native Barcoding Kit 24 V14 (SQK-NBD114.24), and sequenced on an Oxford Nanopore Technologies R10.4.1 flow cell followed by basecalling with Dorado v0.8.1 (https://github.com/nanoporetech/dorado/tree/release-v0.8) with super accuracy. Genome assemblies were created with Autocycler v0.2.1 [37] with 4 read subsets at 25× minimum depth and using the assemblers Canu v2.2 [38], Flye v2.9.5 [39], Miniasm v0.3 [40], NECAT v0.0.1 [41], NextDenovo v2.5.2 [42], and Raven v1.8.3 [43]. For each pair of strains, reads from the *gyrB*^429N^ mutant were mapped to the de novo assembly from the *gyrB*^429D^ parent assembly with SAMtools v1.21 [26] and Minimap2 v2.28 [44]. Variants between each pair of *gyrB*^429D^ and *gyrB*^429N^ strains were identified using Pilon v1.24 [25] with a minimum alignment mapping quality of 20 and minimum depth of 10. The resulting VCF was filtered to keep only variants with an allele frequency >0.9 and a depth >5. Assemblies were annotated with Prokka v1.14.6 [45]. Basecalled reads were uploaded to the NCBI SRA and are available at PRJNA1368854. All variants detected in *gyrB*^D429N^ transformants compared with their parent strains are summarized in Supplementary Table 2.

### Antibiotic Susceptibility Testing

Antibiotic susceptibility testing was performed on GCB-K agar via Etest (BioMerieux) for ciprofloxacin or agar dilution for zoliflodacin and gepotidacin. All MIC results reported are the mean of 3 independent experiments.

### Measurement of Growth and Competitive Fitness of *gyrB* Variants

Growth from overnight cultures of each strain and its *gyrB*^D429N^ derivative on GCB-K plates were suspended in antibiotic-free liquid GCP medium with Kellogg's supplement, diluted to an optical density (OD) of 0.1 (600 nm), and grown for 8 hours at 37°C with 5% CO_2_ with shaking. OD_600_ of each strain was measured at 2, 4, 6, and 8 hours. At every timepoint, cultures were serially diluted and plated on GCB-K agar plates to measure colony forming units (CFUs).

For competition assays, kanamycin resistance was introduced into each strain in the panel of clinical strains and each *gyrB*^D429N^ derivative by electroporation with pDR53, which integrates an *aphA3* marker onto the chromosome between *lctP* and *aspC* [36]. Transformants were selected on GCB-K agar supplemented with 70 µg/mL kanamycin. In pairwise competition experiments, paired strains (1 kanamycin-sensitive and 1 kanamycin-resistant strain) were mixed at a ratio of 1:1 by OD_600_, diluted to OD_600_ ∼0.1, and cocultured in antibiotic-free GCP media with Kellogg's supplement for 8 hours. At each timepoint, cultures were serially diluted and plated on GCB-K agar and GCB-K agar with 70 µg/mL kanamycin. Plates were incubated overnight at 37°C 5% CO_2_. Colonies on each plate were quantified, and the competitive index was calculated at each timepoint as (*R_t_*/*S_t_*)/(*R_0_*/*S_0_*), where *R_t_* and *S_t_* are the proportions of kanamycin-resistant and kanamycin-sensitive strains, respectively, at time *t* and *R_0_* and *S_0_* are the proportions of kanamycin-resistant and kanamycin-sensitive strains at time 0. A competitive index value of 1 indicates equal fitness between strains, >1 indicates the kanamycin-resistant mutant is more fit than the parental strain, and <1 indicates the mutant is less fit. Statistical analysis of competitive index measurements was performed using an unpaired 2-sided Student's *t-test*. All competition experiments were performed by competing unmanipulated, kanamycin-susceptible clinical strains against kanamycin-marked versions of their isogenic *gyrB*^D429N^ derivatives (Figure 3) as well as by competing kanamycin-marked parental strains against kanamycin-susceptible *gyrB*^D429N^ strains (Supplementary Figure 4) to ensure that the kanamycin marker did not contribute to the fitness differences reported here.

## RESULTS

### *gyrB* ^429N^ Consistently Increases Zoliflodacin Resistance but Exerts Strain-Specific Effects on Gepotidacin and Ciprofloxacin Susceptibility

**Figure 1.**
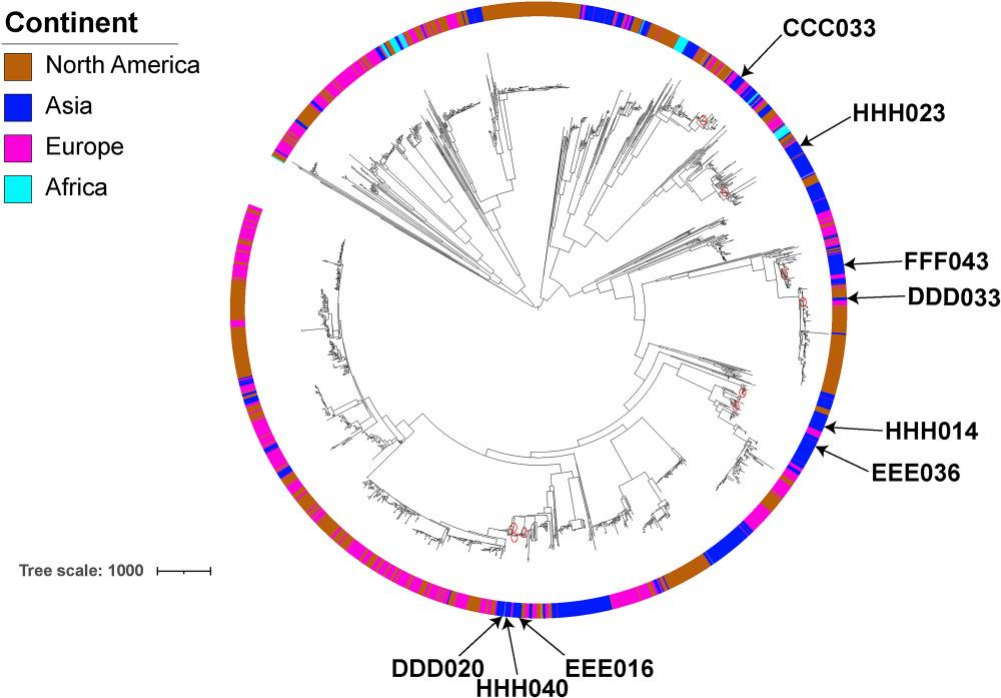
Phylogenetic tree of *N. gonorrhoeae* clinical strains (n = 2800). Tree scale represents the recombination-adjusted number of single-nucleotide polymorphisms. Strains used in this study are marked by arrows. The outer ring represents continents (North America, Asia, Europe, Africa) of the origin of the strains.

To determine the effects of *gyrB*^D429N^ on antimicrobial susceptibility across genetic backgrounds, we introduced this mutation into each of 9 *N. gonorrhoeae* clinical strains, representing common lineages (Figure 1, Supplementary Table 1). These were clinical isolates sampled from the genetic diversity of lineages circulating in Asia, with particular attention to representing important alleles that define ciprofloxacin-resistance genotypes such as *gyrA*^91F, 95G/A^ and *parC* allelic variation at codon positions 86, 87, and 91. The isolates analyzed in this study were selected from Asia because of the high prevalence of ciprofloxacin resistance [3] and the prevalence of ceftriaxone resistance [4], such that we anticipate this location might see early use of zoliflodacin. In each strain, *gyrB*^D429N^ increased zoliflodacin MICs 16- to 32-fold, confirming the ability of this substitution to confer zoliflodacin resistance independent of background (Table 1).

**Table 1.** Zoliflodacin, Gepotidacin, and Ciprofloxacin MICs of *N. gonorrhoeae* Clinical Strains and Their *gyrB*^D429N^ Variants. Dashes (-) Denote Unchanged Alleles From the Parental Strain in Each *gyrB*^D429N^ Variant

Strain	*gyrA*		*parC*			Zoliflodacin	Gepotidacin	Ciprofloxacin
	S91	D95	D86	S87	E91	MIC (µg/mL)	MIC (µg/mL)	MIC (µg/mL)
HHH040	F	G	D	R	E	0.0625	0.25	≥32
HHH040 *gyrB*^D429N^	-	-	-	-	-	2	0.25	4
EEE016	F	G	D	R	E	0.0625	0.25	4
EEE016 *gyrB*^D429N^	-	-	-	-	-	2	0.25	4
DDD020	F	A	D	R	E	0.0625	0.5	≥32
DDD020 *gyrB*^D429N^	-	-	-	-	-	2	0.5	≥32
EEE036	F	A	D	R	E	0.032	0.25	16
EEE036 *gyrB*^D429N^	-	-	-	-	-	0.5	0.25	8
HHH014	F	A	D	S	E	0.032	0.25	4
HHH014 *gyrB*^D429N^	-	-	-	-	-	1	0.25	4
FFF043	F	A	D	S	E	0.016	0.25	0.38
FFF043 *gyrB*^D429N^	-	-	-	-	-	0.5	1	0.125
CCC033	F	A	N	S	E	0.032	1	2
CCC033 *gyrB*^D429N^	-	-	-	-	-	1	32	0.5
DDD033	F	A	N	S	E	0.032	1	8
DDD033 *gyrB*^D429N^	-	-	-	-	-	1	32	1.5
HHH023	F	A	D	N	K	0.0625	1	≥32
HHH023 *gyrB*^D429N^	-	-	-	-	-	1	1	≥32

In 6 out of 9 strains, *gyrB*^D429N^ did not affect gepotidacin resistance, as expected. However, increased gepotidacin MICs were observed in 3 strains: CCC033, DDD033 and FFF043. The *gyrB*^D429N^ substitution resulted in a 32-fold MIC increase in CCC033 and DDD033 (from 1 to 32 µg/mL) and a 4-fold MIC increase in FFF043 (from 0.25 to 1 µg/mL).

CCC033 and DDD033 harbor the *parC*^D86N^ mutation, previously reported to contribute to gepotidacin resistance when in combination with *gyrA*^A92T^. However, Sanger sequencing confirmed that the zoliflodacin- and gepotidacin-resistant *gyrB*^D429N^ derivatives of these strains did not spontaneously acquire the *gyrA*^A92T^ mutation, indicating that the observed MIC increases occurred independently of this canonical resistance pathway.

In contrast, FFF043 lacks variants in the *parC* variants associated with quinolone resistance, indicating other sites may also contribute to zoliflodacin/gepotidacin cross-resistance in certain strain backgrounds (Table 1).

The effects of *gyrB*^D429N^ on ciprofloxacin susceptibility were variable. Five strains (EEE016, DDD020, EEE036, HHH014 and HHH023) maintained ciprofloxacin resistance in the presence of *gyrB*^D429N^. In the remaining 4 strains, ciprofloxacin MICs were reduced by at least 2-fold and in one case as much as ≥8-fold (HHH040) (Table 1). The differential effect of *gyrB*^D429N^ on ciprofloxacin MICs does not correlate with *gyrA* or *parC* genotypes.

### Structural Modeling Shows Close Proximity Between *gyrB*^429^ and *gyrA*^92^, Site of a Canonical Gepotidacin-Resistance Mutation

To understand the mechanism by which *gyrB*^D429N^ can increase gepotidacin MICs, we created a structural model of the *N. gonorrhoeae* GyrA-GyrB heterodimer. The predicted complex (Supplementary Figure 1*A* and 1*B*) showed that GyrA residues 91, 92, and 95 associated with resistance to ciprofloxacin and gepotidacin cluster along the same surface. On the GyrB subunit, zoliflodacin resistance-associated residues 429, 450, and 467 are positioned along a contiguous region.

**Figure 2.**
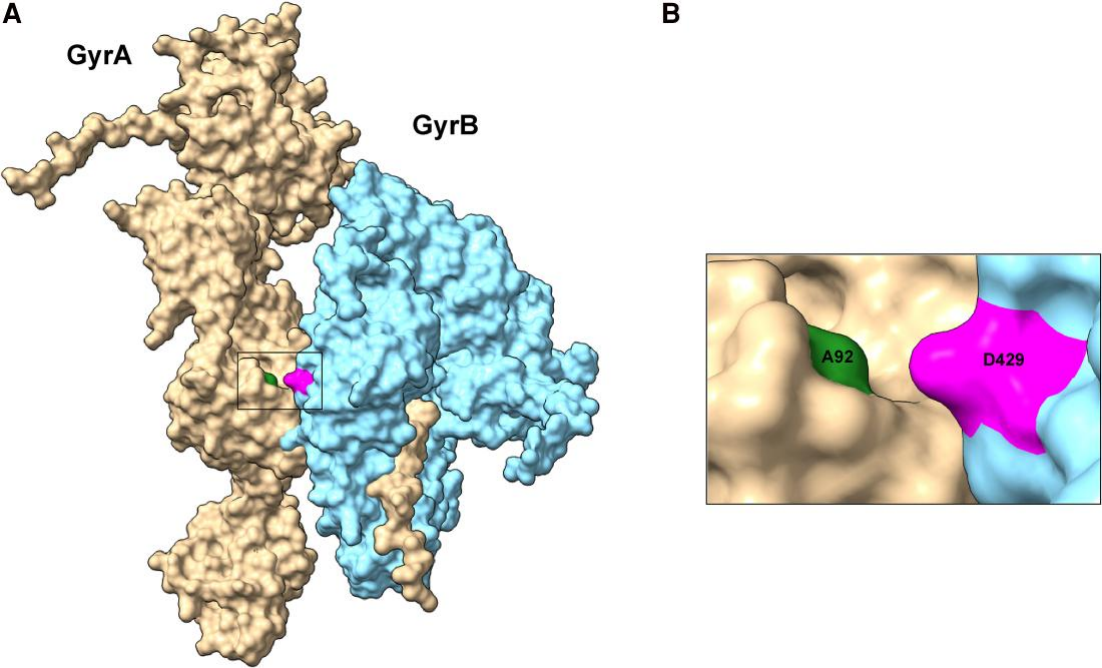
Spatial proximity of GyrA^92^ and GyrB^429^ in the gyrase complex. *A*, ColabFold predicted structure of GyrA (light brown) and GyrB (light blue) docked into a heterodimeric complex. *B*, Magnified view of the GyrA-GyrB interface highlighting residues GyrA^A92^ (green) and GyrB^D429^ (magenta).

Figure 2 *A* and 2*B* provides a localized structural representation highlighting the spatial proximity between GyrA^92^ and GyrB^429^ within the topoisomerase complex. Supplementary Figure 1 provides a broader view of the inhibitor-binding cleft and the amino acid residues in GyrA and GyrB previously implicated in resistance. GyrA^92^, the position at which gepotidacin-resistance mutations were observed following treatment failure in the Phase 2 clinical trial, lies in close spatial proximity to GyrB^429^ (Figure 2*A* and 2*B*). This spatial arrangement suggests that the *gyrB*^D429N^ mutation may alter gepotidacin binding via a similar mechanism to the known resistance mutation *gyrA*^A92T^. Together, these observations support a model in which substitutions at *gyrB*^429^ can influence the local inhibitor-binding environment shared with *gyrA*^92^, providing a rationale for the observed increases in gepotidacin MICs.

### Strain-dependent Fitness Costs of *gyrB*^D429N^ in Ciprofloxacin-Resistant Clinical Strains

**Figure 3.**
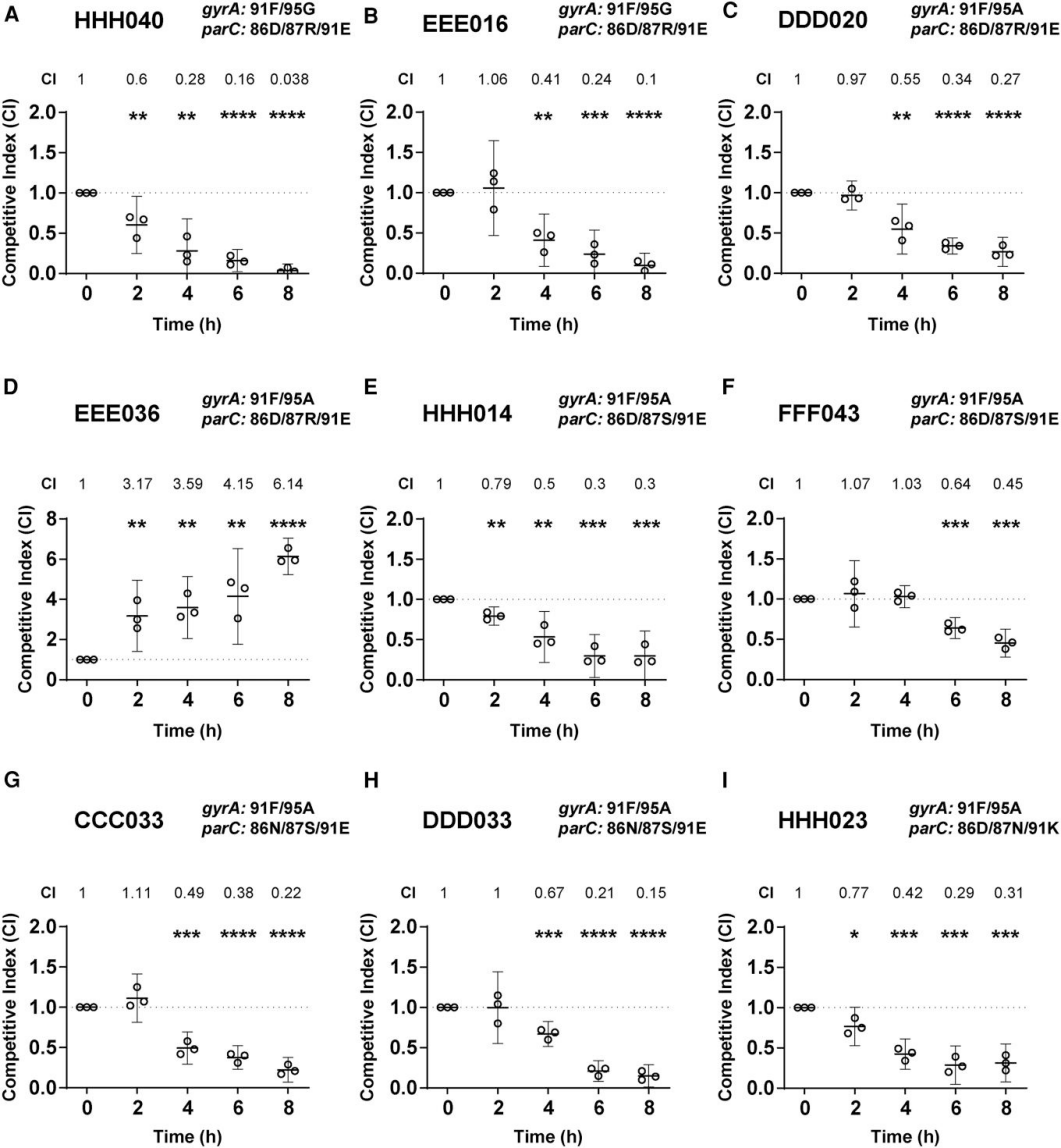
Relative fitness of the *gyrB*^D429N^ mutant in 9 clinical strains backgrounds. *Y*-axes show the competitive index (CI) of each *gyrB*^D429N^ mutant relative to its parental strain during competitive growth in vitro. In all cases, the *gyrB*^D429N^ mutant carried a kanamycin marker and was cocultured with its unmarked parental strain. *A*, HHH040: *P* = .009, .002, <.0001, <.0001, respectively for 2, 4, 6, and 8 h. *B*, EEE016: *P* = .7, .001, .0004, <.0001, respectively for 2, 4, 6, and 8 h. *C*, DDD020: *P* = .47, .003, <.0001, <.0001, respectively for 2, 4, 6, and 8 h. *D*, EEE036: *P* = .006, .002, .005, <.0001, respectively for 2, 4, 6, and 8 h. *E*, HHH014: *P* = .001, .003, .0003, .0006, respectively for 2, 4, 6, and 8 h. *F*, FFF043: *P* = .53, .4, .0003, .0002, respectively for 2, 4, 6, and 8 h. *G*, CCC033: *P* = .18, .0004, <.0001, <.0001, respectively for 2, 4, 6, and 8 h. *H*, DDD033: *P* = .98, .0008, <.0001, <.0001, respectively for 2, 4, 6, and 8 h. *I*, HHH023: *P* = .01, .002, .0002, .0002, respectively for 2, 4, 6, and 8 h. n = 3, representative of 3 independent experiments performed in absence of any antibiotic pressure. Error bars represent mean with 95% confidence interval. Statistically significant differences in competitive indices compared with time 0 were analyzed using an unpaired Student's *t-test*, indicated *P ≤ .05, **P ≤ .01, ***P ≤ .001, and ****P ≤ .0001.

To evaluate the fitness impact of *gyrB*^D429N^, we quantified in vitro growth of each strain and its *gyrB*^D429N^ derivative in antibiotic-free gonococcal medium in monoculture using both OD_600_-based growth measurements and viable CFU assays (Supplementary Figures 2 and 3) and in pairwise competition assays (Figure 3; Supplementary Figure 4). CFU enumeration directly quantifies viable bacteria and therefore provides a more precise assessment of growth impairments associated with *gyrB*^D429N^ mutation. *gyrB*^D429N^ transformants in HHH040 and HHH014 showed growth impairment in both OD_600_ (Supplementary Figure 2*A* and 2*E*) and CFU-based assays (Supplementary Figure 3*A*, 3*D* and 3*E*). *gyrB*^D429N^ transformants in EEE016, EEE036, FFF043, CCC033 and DDD033 showed no substantial growth defects compared to their parental strains by both OD_600_ (Supplementary Figure 2*B*, 2*D* and 2*F*–H) and CFU measurements (Supplementary Figure 3*B*, 3*D* and 3*F*–*H*). However, DDD020 and HHH023 *gyrB*^D429N^ mutants showed significant reduction in viable counts beginning at 4 hours by CFU plating but not by OD_600_-based growth measurements (Supplementary Figures 2*C*, 2*I* and 3*C*, 3*I*). Since OD_600_ measurements do not distinguish live from nonviable bacteria and can be influenced by changes in cell morphology, aggregation, and vesicle formation, these findings suggest that OD-based assays alone may underestimate subtle growth defects associated with *gyrB*^D429N^ in some strains.

Introduction of *gyrB*^D429N^ imposed a fitness cost in most of the strains (Figure 3, Supplementary Figure 4), although in some cases the fitness cost of *gyrB*^D429N^ was sufficiently modest that virtually no growth defect was detected in individual growth curves (Supplementary Figures 2 and 3). The strongest fitness costs were observed in HHH040 and EEE016, both of which carry *gyrA*^91F, 95G^ and *parC*^S87R^. In all but one other strain, *gyrB*^D429N^ conferred a fitness cost that was significant but less extreme. However, in the strain EEE036, the introduction of *gyrB*^D429N^ resulted in a significant fitness advantage (Figure 3*D*; Supplementary Figure 4*D*). Whole genome sequencing confirmed that no off-target mutations occurred in the EEE036 *gyrB*^D429N^ strain, with only the target *gyrB*^D429N^ mutation and no other variants detected in the complete genome of this mutant compared with its isogenic parent strain (Supplementary Table 2). Interestingly, EEE036 carries the same combination of *gyrA*^91F/95A^ and *parC*^S87R^ alleles found in DDD020, in which the *gyrB*^D429N^ mutation incurred a substantial fitness cost (Figure 3*C*; Supplementary Figure 4*C*). The unexpected fitness benefit of *gyrB*^D429N^ in this strain is therefore not a result of epistasis with these other replisome components, suggesting that EEE036 harbors another, as-yet unidentified genetic variant that accommodates the *gyrB*^D429N^ mutation. Whole genome sequencing did not detect secondary mutations in the *gyrB*^D429N^ mutants that raised concern for contributing to antimicrobial resistance. Three strains had no other mutations, and others exhibited expected genetic changes attributable to high-frequency phase variation at sites such as *opa*, *pilE*, and *modA* or changes in mobile genetic elements, sites not expected to impact antimicrobial susceptibility or fitness phenotypes (Supplementary Table 2). All pairwise competition results were consistent with the reciprocal experiment in which the kanamycin marker was carried by the opposite strain, thus ensuring that the results are not influenced by a fitness effect conferred by the resistance marker (Supplementary Figure 4*A*–*I*).

## DISCUSSION

Following the recent approval of zoliflodacin and gepotidacin for uncomplicated gonorrhea by the United States FDA, it is crucial to strategically deploy these drugs to maximize their useful therapeutic lifespan and to delay the emergence of resistance as much as possible. Understanding the evolutionary pathways to resistance for these drugs in diverse strain backgrounds is crucial for informing their recommended use and the surveillance necessary to monitor their continuing effectiveness. This is particularly important for strains circulating in Asia, as high resistance to existing drugs in this region may cause greater use of new therapeutics.

To this end, we examined the effects of the zoliflodacin-resistance mutation *gyrB*^D429N^ in a panel of diverse clinical strains from Asia that includes genetic variation in key replisome components targeted by ciprofloxacin. The *gyrB*^D429N^ mutation invariably increased zoliflodacin MIC among these strains, but modulated susceptibility to ciprofloxacin and gepotidacin in a strain-dependent manner.

While we previously reported that *gyrB*^D429N^ increases resistance to ciprofloxacin in a ciprofloxacin-susceptible strain [11], we did not observe increased ciprofloxacin MICs when we introduced this mutation into ciprofloxacin-resistant clinical strains carrying the *gyrA*^91F^ allele (Table 1). On the contrary, the introduction of *gyrB*^D429N^ decreased ciprofloxacin MICs in some of these strains. The genetic variants that drive this background-specific effect have not yet been identified but may elucidate constraints in the evolution of replisome components in response to drug pressure.

Concerningly, *gyrB*^D429N^ conferred both zoliflodacin and gepotidacin resistance in clinical strains that carried the *parC*^D86N^ variant. High-level resistance to gepotidacin in *N. gonorrhoeae* has previously been observed only through a combination of *parC*^D86N^ with *gyrA*^A92T^ [15–17]. The observed requirement for these 2 mutations is consistent with the known mechanism of action of gepotidacin, which targets both the gyrase and the topoisomerase IV complexes of *N. gonorrhoeae* [8]. Our study suggests that *gyrB*^D429N^ can also yield gepotidacin resistance (MIC = 32 µg/mL) in the *parC*^D86N^ background, even in the absence of the canonical *gyrA*^A92T^ mutation. While the *gyrB*^D429N^ mutation in *N. gonorrhoeae* has not previously been implicated in gepotidacin resistance, the package insert for BLUJEPA (gepotidacin) describes otherwise unpublished data suggesting that the homologous aspartic acid residue in *Escherichia coli* and *Klebsiella pneumoniae*, *gyrB*^D426^, “may be important for gepotidacin activity” [46]. Taken together, these results point to a working model in which gepotidacin resistance requires target site mutations at both known drug targets: the *parC*^D86N^ mutation to preserve topoisomerase IV function, and either the *gyrA*^A92T^ or the *gyrB*^D429N^ mutation to preserve gyrase function.

The ability of the zoliflodacin-resistance mutation *gyrB*^D429N^ to confer resistance to gepotidacin raises concern for the emergence of cross-resistant strains. Regions where zoliflodacin may be introduced first due to emerging ceftriaxone resistance also have reported a high prevalence of *parC*^D86N^: 18.9% in Vietnam [31], 36.7% in Cambodia [32], and 46.5% in Thailand [23]. Cross-resistance would be expected to shorten the duration of the new drugs’ clinically useful lifespans [47, 48].

The fitness consequences of resistance mutations play a major role in determining how quickly resistance arises and spreads. We found that the fitness effects of *gyrB*^D429N^ varied with genetic background, ranging from deleterious to advantageous. While *gyrB*^D429N^ imposed a fitness cost in most clinical strains we tested, the fitness advantage it conferred in strain EEE036 demonstrates that this resistance mutation is not universally costly. The translation of in vitro fitness to fitness within the context of human infections is uncertain, although other studies have shown a correlation [36, 49]. If this unexpected fitness benefit of *gyrB*^D429N^ translates to in vivo settings, this observation indicates that some zoliflodacin-resistant strains could persist and spread even in the absence of drug pressure and further suggests that resistance could be similarly stabilized in other lineages by the evolution of compensatory mutations.

Our results have several additional implications. First, the susceptibility and fitness effects of *gyrB*^D429N^ are modulated by genetic background, indicating that additional factors remain to be discovered. Second, standard surveillance approaches that track only individual loci (eg, *gyrB*) will likely be insufficient for monitoring resistance phenotypes. Third, early recognition of background-dependent resistance emergence could allow for regionally tailored treatment guidelines, preventing widespread therapeutic failure [50]. Fourth, while we focused here on the *gyrB*^D429N^ mutation, other *gyrB* mutations located in a similar region of the protein can also contribute to zoliflodacin-resistance, either directly [12] or as stepping-stone mutations [10, 13]. The propensity of these mutations to confer cross-resistance to other drug classes should similarly be determined in a variety of strain backgrounds.

Finally, while early in vitro and clinical experiences with the 2 new drugs identified some resistance mutations, our findings indicate that other, in this case cross-resistance conferring, mutations may arise. More comprehensive analysis and vigilant surveillance for phenotypic resistance as the new drugs are rolled out will be important to inform optimal clinical use and to maximize their public health benefit.

## Supplementary Material

jiag174_Supplementary_Data

## Supplementary Data

Supplementary materials are available at *The Journal of Infectious Diseases* online (http://jid.oxfordjournals.org/). Supplementary materials consist of data provided by the author that are published to benefit the reader. The posted materials are not copyedited. The contents of all supplementary data are the sole responsibility of the authors. Questions or messages regarding errors should be addressed to the author.
