# Supplementary material for: Genetic Background Modulates Zoliflodacin and Gepotidacin Cross-Resistance and Fitness in Neisseria gonorrhoeae
Source: J Infect Dis. 2026 Mar 19;234(1):e131–40. doi: 10.1093/infdis/jiag174 (PMC13061509; doi:10.1093/infdis/jiag174)
Supplement: jiag174_Supplementary_Data [file jiag174_supplementary_data.zip › JID84630_revision_Suppl_Clean_03112026.docx]

**Genetic background modulates zoliflodacin and gepotidacin cross-resistance and fitness in *Neisseria gonorrhoeae***

Aditi Mukherjee^1^, Sofia OP Blomqvist^1^, David Helekal^1^, Apabrita A Das^2,3^, Samantha G Palace^1,4^, Yonatan H Grad^1,4,5^

1. Department of Immunology and Infectious Diseases, Harvard T. H. Chan School of Public Health, Boston, Massachusetts, USA
2. Cardiovascular Medicine Division, Brigham and Women’s Hospital, Boston, Massachusetts, USA
3. Harvard Medical School, Boston, Massachusetts, USA
4. Co-senior authors
5. Corresponding author

**Running title:** Zoliflodacin and gepotidacin cross-resistance

**Corresponding author’s contact information:** Yonatan Grad, Department of Immunology and Infectious Diseases, Harvard T.H. Chan School of Public Health, Room 715, Building 1, 655 Huntington Ave, Boston, MA 02115, USA; [ygrad@hsph.harvard.edu](mailto:ygrad@hsph.harvard.edu)

**Supplementary Files**

**Supplementary Table 1:** Plasmids, *N. gonorrhoeae* strains and primers used in this study

| **Plasmids** | **Description** | **Reference** |
| --- | --- | --- |
| pDR53 | A derivative of pDR1 without Lac promoter and the regulatory elements + added multi cloning site (MCS) | (1) |

| **Strains** | **NG-STAR** | **MLST** | **Genotype** | **Reference** |
| --- | --- | --- | --- | --- |
| HHH040 | 5093 | 10314 | *gyrA*^91F/95G^, *parC*^S87R^ | (2) |
| HHH040 *gyrB*^D429N^ | NA | NA | HHH040, *gyrA*^91F/95G^, *parC*^S87R^, *gyrB*^D429N^ | This study |
| EEE016 | 127 | 10314 | *gyrA*^91F/95G^, *parC*^S87R^ | (2) |
| EEE016 *gyrB*^D429N^ | NA | NA | EEE016, , *gyrA*^91F/95G^, *parC*^S87R^, *gyrB*^D429N^ | This study |
| DDD020 | 1148 | 1901 | *gyrA*^91F/95A^, *parC*^S87R^ | (2) |
| DDD020 *gyrB*^D429N^ | NA | NA | DDD020, *gyrA*^91F/95A^, *parC*^S87R^, *gyrB*^D429N^ | This study |
| EEE036 | 73 | 7822 | *gyrA*^91F/95A^, *parC*^S87R^ | (2) |
| EEE036 *gyrB*^D429N^ | NA | NA | EEE036, *gyrA*^91F/95A^, *parC*^S87R^, *gyrB*^D429N^ | This study |
| HHH014 | 3306 | 1583 | *gyrA*^91F/95A^, *parC*^D86/S87/E91^ | (2) |
| HHH014 *gyrB*^D429N^ | NA | NA | HHH014, *gyrA*^91F/95A^, *parC*^D86/S87/E91^, *gyrB*^D429N^ | This study |
| FFF043 | NA  Untyped *parC* | 18456 | *gyrA*^91F/95A^, *parC*^D86/S87/E91^ | (2) |
| FFF043 *gyrB*^D429N^ | NA | NA | FFF043, , *gyrA*^91F/95A^, *parC*^D86/S87/E91^, *gyrB*^D429N^ | This study |
| CCC033 | 427 | 8143 | *gyrA*^91F/95A^, *parC^D^*^86N^ | (2) |
| CCC033 *gyrB*^D429N^ | NA | NA | CCC033, *gyrA*^91F/95A^, *parC^D^*^86N^, *gyrB*^D429N^ | This study |
| DDD033 | 2477 | 7363 | *gyrA*^91F/95A^, *parC^D^*^86N^ | (2) |
| DDD033 *gyrB*^D429N^ | NA | NA | DDD033, *gyrA*^91F/95A^, *parC^D^*^86N^, *gyrB*^D429N^ | This study |
| HHH023 | 567 | 1588 | *gyrA*^91F/95A^, *parC*^S87N/E91K^ | (2) |
| HHH023 *gyrB*^D429N^ | NA | NA | HHH023, *gyrA*^91F/95A^, *parC*^S87N/E91K^, *gyrB*^D429N^ | This study |

| **Primers** | **Sequence (Annealing)** | **Description** |
| --- | --- | --- |
| DR_395 | ATTAAATGCGTTTAAACATGCTCTAGAACGCGTCGACGCT | Forward to amplify Kan^R^ derivative of pKH37 from pDRE1 |
| DR_396 | TGTTTAAACGCATTTAATTAAGAACGGATGGTTCTTGTTG | Reverse to amplify Kan^R^ derivative of pKH37 from pDRE1 |
| AM_1 | ATGCCGTCTGAAATGCGCAATGCAATGGAACG | Forward to amplify *gyrB* allele from *Ng* gDNA |
| AM_2 | GATGACGGTTTTCGCCAACA | Reverse to amplify *gyrB* allele from *Ng* gDNA |

**Supplementary Table 2: Whole genome sequencing analysis of *gyrB*^D429N^ transformants**

| **Mutant** | **Reference** | **Variants** |
| --- | --- | --- |
| HHH040 *gyrB*^D429N^ | HHH040 | *gyrB*^D429N^, one *opa* pentanucleotide repeat variation*, repeat variation in gene of unknown function homologous to NCCP11945 NGK_RS02285 |
| HHH014 *gyrB*^D429N^ | HHH014 | *gyrB*^D429N^, changes in two *opa* genes*, repeat variation in site-specific DNA methyltransferase M.NgoAXII* |
| HHH023 *gyrB*^D429N^ | HHH023 | *gyrB*^D429N^, single nucleotide deletion in intergenic region between IS*110*-family transposase and hypothetical protein homologous to NCCP11945 NGK_RS06655 |
| EEE016 *gyrB*^D429N^ | EEE016 | *gyrB*^D429N^, *pilT* SNP, SNP in a transposase pseudogene, repeat variation in gene of unknown function homologous to NCCP11945 NGK_RS02285, small insertion in *mexA*, changes in one *opa* gene*, repeat variation in site-specific DNA methyltransferase M.NgoAXII* |
| FFF043 *gyrB*^D429N^ | FFF043 | *gyrB*^D429N^, *pilE* SNP, repeat variation in site-specific DNA methyltransferase M.NgoAXII* |
| DDD020 *gyrB*^D429N^ | DDD020 | *gyrB*^D429N^, no others |
| DDD033 *gyrB*^D429N^ | DDD033 | *gyrB*^D429N^, one *opa* pentanucleotide repeat variation*, repeat variation in site-specific DNA methyltransferase M.NgoAXII* |
| CCC033 *gyrB*^D429N^ | CCC033 | *gyrB*^D429N^, no others |
| EEE036 *gyrB*^D429N^ | EEE036 | *gyrB*^D429N^, no others |

* High frequency variation at *opa* and *modA* (encoding M.NgoXII) loci is known to occur via changes in repeat copy number (3, 4) and via intergenic recombination (for *opa*) (5). Variation in opa and *modA* is known to influence adhesion (e.g., reviewed in (6)) and epigenetic programming (4) of *N. gonorrhoeae*, respectively. Neither *opa* nor *modA* phase variation in the *gyrB*^D429N^ derivatives we studied here correlated with changes in resistance (**Table 1**) or fitness (**Figure 3, Supplementary Figure 4**).

**Supplementary Figure 1:** (A) ColabFold predicted structure of wildtype GyrA (light brown) and wildtype GyrB (light blue) docked into a heterodimeric complex. (B) Magnified view of the GyrA-GyrB interface highlighting residues GyrA^S91^ (orange), GyrA^A92^ (green), GyrA^D95^ (purple) and GyrB^D429^ (magenta), GyrB^K450^ (blue), GyrB^S467^ (yellow).

**Supplementary Figure 2:** *In vitro* growth kinetics of strains with and without *gyrB*^D429N^, measured by spectrophotometry. All strains (black) and their isogenic *gyrB*^D429N^ mutants (red) were cultured separately in liquid GCP media supplemented with Kellogg’s supplement at a starting absorbance reading at 600nm (OD_600_) = 0.1. OD_600_ readings at 0, 2, 4, 6 and 8 hours timepoint are plotted. (A) HHH040 and HHH040 *gyrB*^429N^. p = 0.44, 0.2, 0.002, 0.03, respectively for 2, 4, 6 and 8 hours. (B) EEE016 and EEE016 *gyrB*^429N^. (C) DDD020 and DDD020 *gyrB*^429N^. (D) EEE036 and EEE036 *gyrB*^429N^. p = 0.002, 0.001, 0.02, 0.11, respectively for 2, 4, 6 and 8 hours. (E) HHH014 and HHH014 *gyrB*^429N^. p = 0.005, 0.003, 0.006, 0.002, respectively for 2, 4, 6 and 8 hours. (F) FFF043 and FFF043 *gyrB*^429N^. (G) CCC033 and CCC033 *gyrB*^429N^. (H) DDD033 and DDD033 *gyrB*^429N^. (I) HHH023 and HHH023 *gyrB*^429N^. n = 3, representative of three independent experiments. Error bars represent SDs between three biological replicates. Statistical significance was determined by unpaired two-sided Student’s *t-test* and are indicated *p ≤ 0.05 and **p ≤ 0.01.

**Supplementary Figure 3:** *In vitro* growth kinetics of strains with and without *gyrB*^D429N^, measured by dilution plating. All strains (black) and their isogenic *gyrB*^D429N^ mutants (red) were cultured separately in liquid GCP media supplemented with Kellogg’s supplement at a starting absorbance reading at 600nm (A_600_) = 0.1. Dilutions were plated on GCB-K plates at 0, 2, 4, 6 and 8 hours, colonies were counted after overnight growth, and CFUs were calculated. (A) HHH040 and HHH040 *gyrB*^429N^. p = 0.14, 0.01, <0.0001, <0.0001, respectively for 2, 4, 6 and 8 hours. (B) EEE016 and EEE016 *gyrB*^429N^. p = 0.7, 0.76, 0.73, <0.0001, respectively for 2, 4, 6 and 8 hours. (C) DDD020 and DDD020 *gyrB*^429N^. p = 0.23, 0.03, 0.01, 0.001, respectively for 2, 4, 6 and 8 hours. (D) EEE036 and EEE036 *gyrB*^429N^. p = 0.0009, 0.001, 0.24, 0.25, respectively for 2, 4, 6 and 8 hours. (E) HHH014 and HHH014 *gyrB*^429N^. p = 0.02, 0.0008, 0.07, 0.0001, respectively for 2, 4, 6 and 8 hours. (F) FFF043 and FFF043 *gyrB*^429N^ (p value is not significant at all timepoints). (G) CCC033 and CCC033 *gyrB*^429N^. p = 0.31, 0.48, 0.27, 0.002, respectively for 2, 4, 6 and 8 hours. (H) DDD033 andDDD033 *gyrB*^429N^. p = 0.07, <0.0001, 0.85, 0.63, respectively for 2, 4, 6 and 8 hours. (I) HHH023 and HHH023 *gyrB*^429N^. p = 0.002, 0.42, 0.003, 0.01, respectively for 2, 4, 6 and 8 hours. n = 3, representative of three independent experiments. Error bars represent SDs between three biological replicates. Statistical significance was determined by unpaired two-sided Student’s *t-test* and are indicated *p ≤ 0.05, **p ≤ 0.01, ***p ≤ 0.001 and ****p ≤ 0.0001.

**Supplementary Figure 4:** Relative fitness of clinical strains compared to their isogenic *gyrB*^D429N^ mutants in 9 clinical strains backgrounds. Y-axes show the Competitive Index (CI) of each parental strain relative to its *gyrB*^D429N^ mutant during competitive growth *in vitro*. In all cases, the parental strain carried a kanamycin marker and was cocultured with its unmarked *gyrB*^D429N^ mutant. (A) HHH040: p = 0.01, 0.005, <0.0001, 0.0001, respectively for 2, 4, 6 and 8 hours. (B) EEE016: p = 0.9, 0.04, 0.02, 0.001, respectively for 2, 4, 6 and 8 hours. (C) DDD020: p = 0.92, 0.001, 0.0003, 0.004, respectively for 2, 4, 6 and 8 hours. (D) EEE036: p = 0.004, <0.0001, <0.0001, <0.0001, respectively for 2, 4, 6 and 8 hours. (E) HHH014: p = 0.51, 0.003, 0.002, <0.0001, respectively for 2, 4, 6 and 8 hours. (F) FFF043: p = 0.12, 0.32, 0.04, 0.02, respectively for 2, 4, 6 and 8 hours. (G) CCC033: p = 0.03, 0.02, 0.005, 0.02, respectively for 2, 4, 6 and 8 hours. (H) DDD033: p = 0.79, 0.02, 0.0004, <0.0001, respectively for 2, 4, 6 and 8 hours. (I) HHH023: p = 0.003, 0.01, 0.002, <0.0001, respectively for 2, 4, 6 and 8 hours. n = 3, representative of three independent experiments performed in absence of any antibiotic pressure. Error bars represent mean with 95% confidence interval. Statistically significant differences in competitive indices compared to time 0 were analyzed using an unpaired Student’s *t-test*, indicated *p ≤ 0.05, **p ≤ 0.01, ***p ≤ 0.001 and ****p ≤ 0.0001.

**References**

1. Helekal D, Mortimer TD, Mukherjee A, Gentile G, Van AL, Blomqvist S, et al. Quantifying the real-world impact of antibiotic use and genetic determinants of resistance on gonococcal dynamics. Nat Microbiol. 2026.

2. Bristow CC, Mortimer TD, Morris S, Grad YH, Soge OO, Wakatake E, et al. Whole-Genome Sequencing to Predict Antimicrobial Susceptibility Profiles in Neisseria gonorrhoeae. J Infect Dis. 2023;227(7):917-25.

3. Stern A, Brown M, Nickel P, Meyer TF. Opacity genes in Neisseria gonorrhoeae: control of phase and antigenic variation. Cell. 1986;47(1):61-71.

4. Srikhanta YN, Dowideit SJ, Edwards JL, Falsetta ML, Wu HJ, Harrison OB, et al. Phasevarions mediate random switching of gene expression in pathogenic Neisseria. PLoS Pathog. 2009;5(4):e1000400.

5. Connell TD, Black WJ, Kawula TH, Barritt DS, Dempsey JA, Kverneland K, Jr., et al. Recombination among protein II genes of Neisseria gonorrhoeae generates new coding sequences and increases structural variability in the protein II family. Mol Microbiol. 1988;2(2):227-36.

6. Walker E, van Niekerk S, Hanning K, Kelton W, Hicks J. Mechanisms of host manipulation by Neisseria gonorrhoeae. Front Microbiol. 2023;14:1119834.
