# Supplementary figures and images for: Genetic Background Modulates Zoliflodacin and Gepotidacin Cross-Resistance and Fitness in Neisseria gonorrhoeae
Source: J Infect Dis. 2026 Mar 19;234(1):e131–40. doi: 10.1093/infdis/jiag174 (PMC13061509; doi:10.1093/infdis/jiag174)

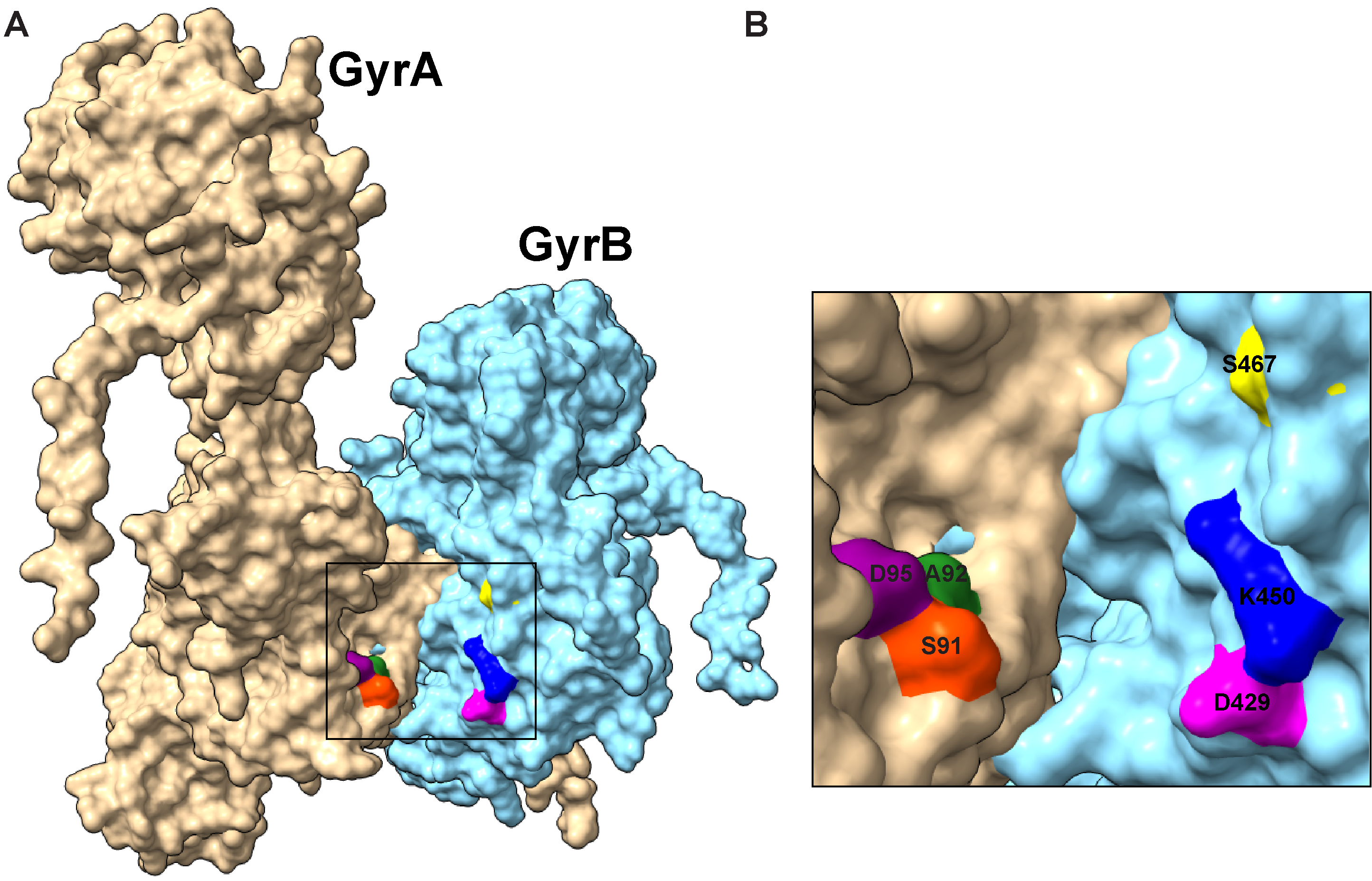

Supplement: jiag174_Supplementary_Data [file jiag174_supplementary_data.zip › Supplementary Figure 1.tif]

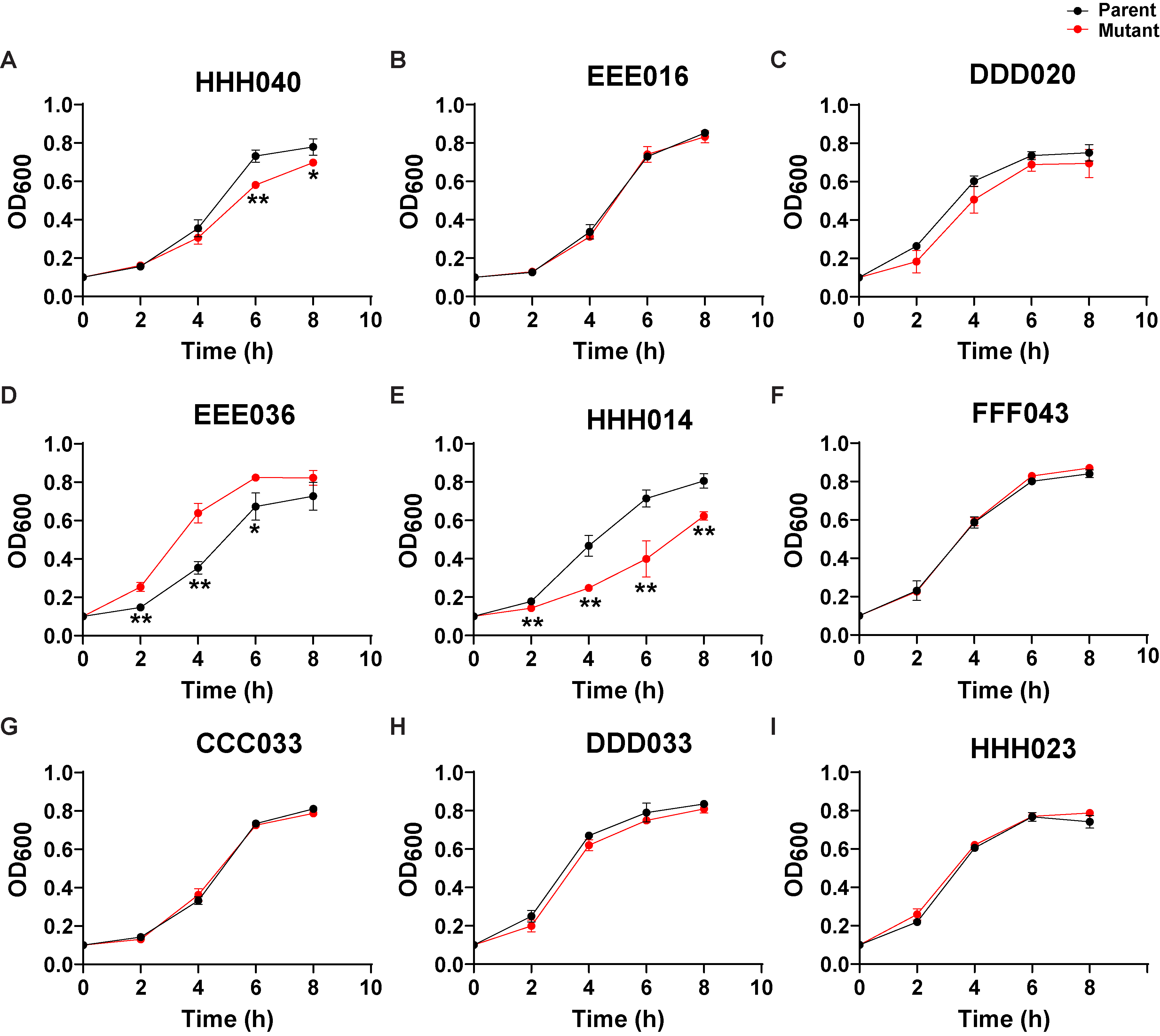

Supplement: jiag174_Supplementary_Data [file jiag174_supplementary_data.zip › Supplementary Figure 2.tif]

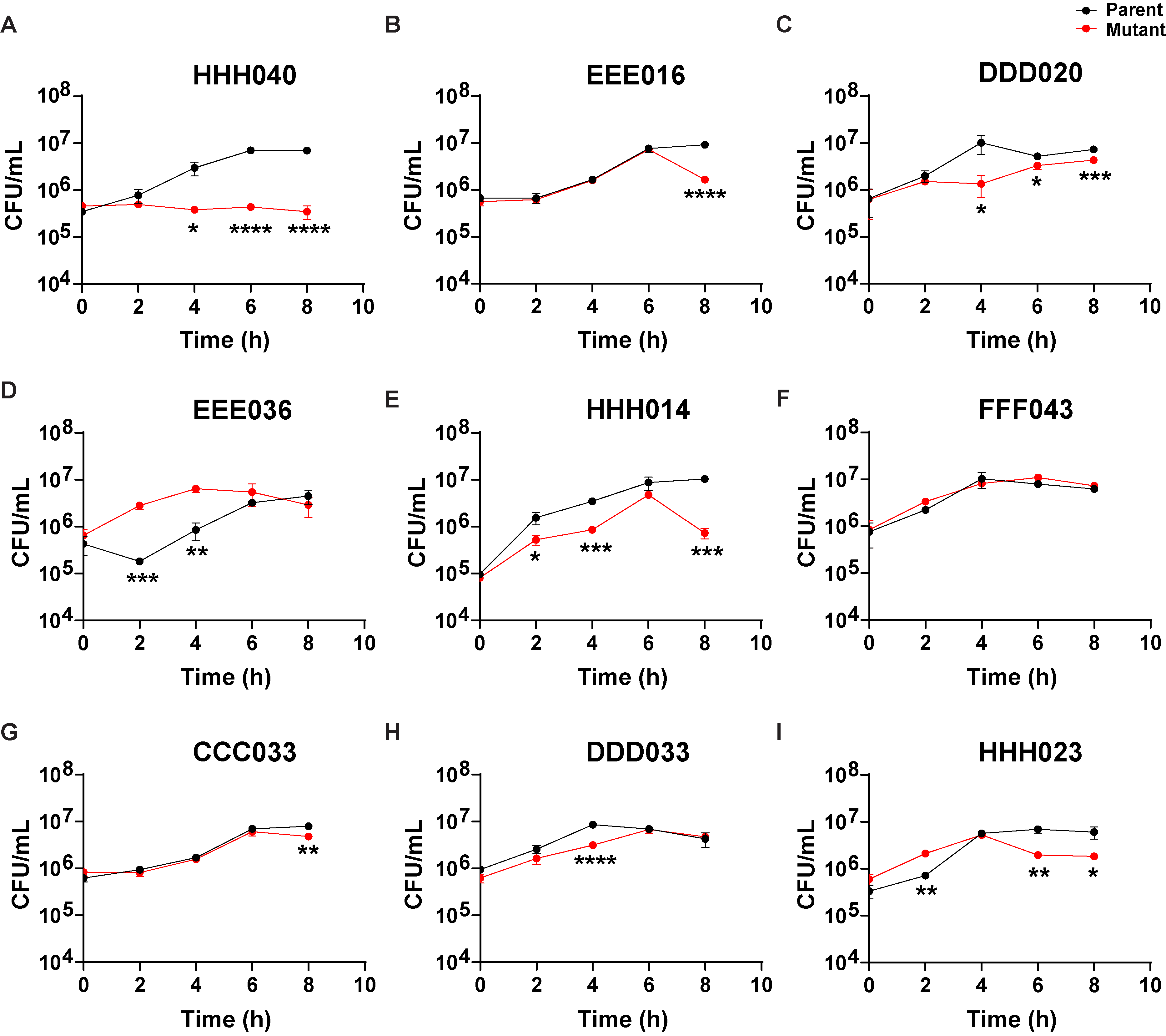

Supplement: jiag174_Supplementary_Data [file jiag174_supplementary_data.zip › Supplementary Figure 3.tif]

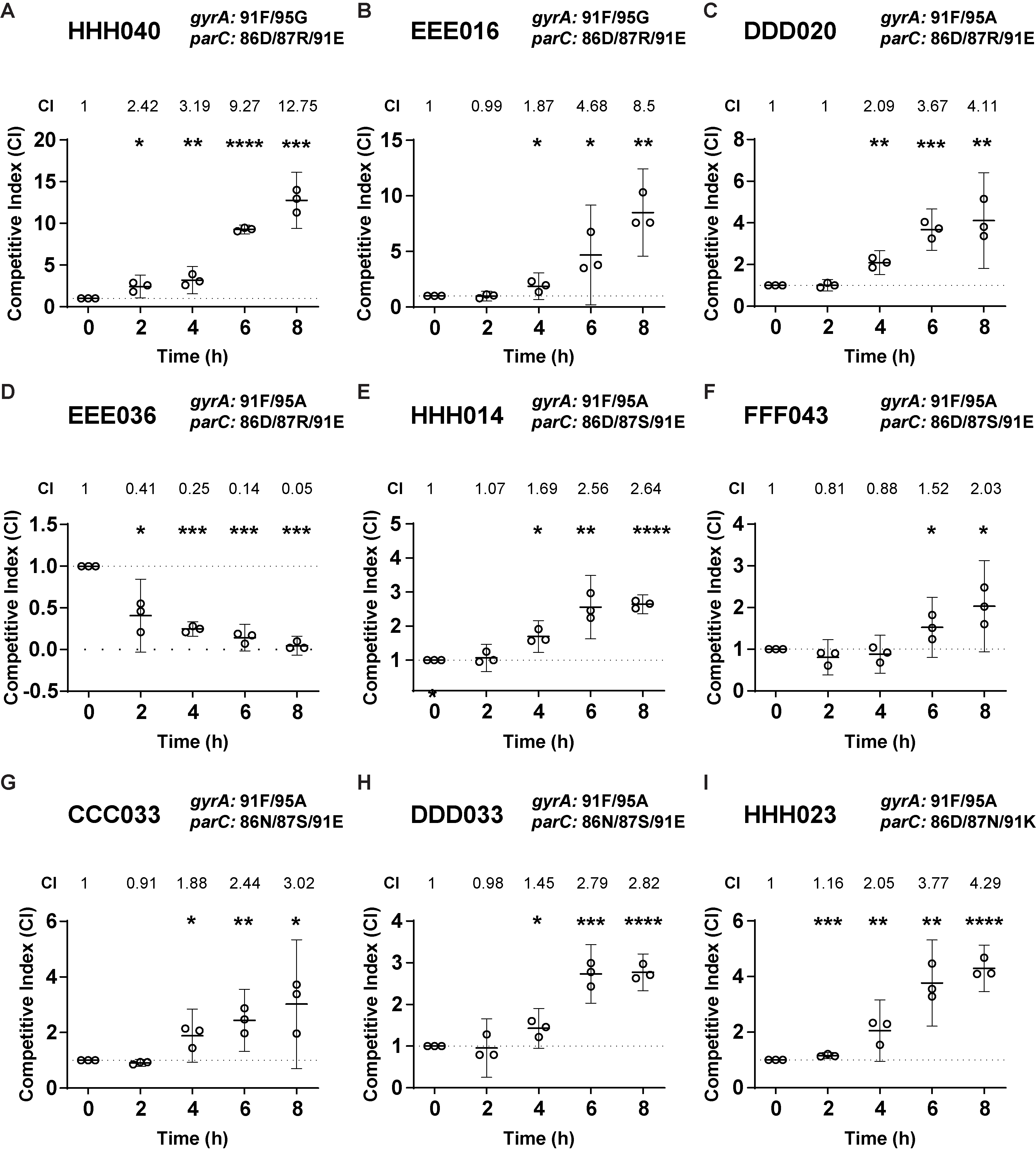

Supplement: jiag174_Supplementary_Data [file jiag174_supplementary_data.zip › Supplementary Figure 4.tif]
